# A Virological Perspective on Cancer

**DOI:** 10.1371/journal.ppat.1005326

**Published:** 2016-02-11

**Authors:** Blossom Damania

**Affiliations:** Lineberger Comprehensive Cancer Center and Department of Microbiology & Immunology, University of North Carolina at Chapel Hill, Chapel Hill, North Carolina, United States of America; University of Florida, UNITED STATES

Cancer has prevailed for millions of years, likely dating back to the very first appearance of multicellular organisms. Some of the first documentations of human cancer can be found in the Code of Hammurabi (1750 BCE) and the ancient Egyptian papyri (1600 BCE). Currently, it is a widely accepted fact that infectious agents are responsible for approximately 20%–25% of the world’s cancer burden. Most cancer-associated infectious agents are oncogenic viruses that are linked to a multitude of different human cancers. The field of tumor virology encompasses the study of all known oncogenic viruses. Human tumor viruses include human papillomavirus (HPV), hepatitis B virus (HBV), hepatitis C virus (HCV), Epstein–Barr virus (EBV), Kaposi’s sarcoma-associated herpesvirus (KSHV), human T-lymphotropic virus (HTLV-1), and Merkel cell polyomavirus (MCV).

Historically, identification of the intricate relationship between viruses and cancer was a landmark discovery that laid the foundation for our understanding of the concepts of modern cancer biology. The study of oncogenic viruses led to seminal insights into the underlying mechanisms of how cancers arise. The field of tumor virology was founded in 1911, when Peyton Rous reported that a cell-free extract (containing Rous sarcoma virus; RSV) injected in chickens could cause tumors. Since then, the study of tumor viruses has led to groundbreaking discoveries in cancer cell biology. Research on tumor viruses gave rise to the concept of cellular oncogenes and tumor suppressors, which was solidified by the identification of oncogenes like Src through studies on RSV, and by the identification of tumor suppressors—e.g., p53 and Rb—from studies of simian virus 40 (SV40). Hence, in addition to being etiologic agents of cancer, viruses have also provided us with the tools to understand the mechanisms that underlie the development of human cancer as well as basic cell biology.

My foray into the world of tumor virology began as a graduate student studying SV40 large T antigen, the viral oncoprotein whose function led scientists to discover the cellular tumor suppressor, p53. As a student, I explored how SV40 large T antigen was able to subvert the transcriptional machinery of human cells and activate a plethora of host and viral genes by acting like a cellular TATA-binding protein (TBP)-associated factor (TAF). I was intrigued by how one viral protein, surrounded by several thousand cellular proteins, could nevertheless commandeer the machinery of the cell. I was also fascinated by the fact that expression of just this one viral protein could transform a perfectly normal cell into a rapidly growing, immortal cancer cell. The ability of oncogenic viruses to exploit cellular processes to their advantage was captivating and prompted me to pursue a postdoctoral fellowship studying another tumor virus, Kaposi’s sarcoma-associated herpesvirus (KSHV), which was discovered in 1994 by Drs. Yuan Chang and Patrick Moore.

KSHV is a gammaherpesvirus that is associated with three different human cancers and has a large genome coding for over 100 different gene products, ranging from proteins to microRNAs and long noncoding RNAs. The genome coding capacity of this herpesvirus ensured that I would have plenty to work on, for decades to come, when I started my own laboratory over 15 years ago at the University of North Carolina at Chapel Hill (UNC-CH).

Today, my laboratory’s research focuses on the intersection of viruses, cancer biology, and immunology. We study how KSHV viral proteins influence cellular signaling pathways associated with cell proliferation, in order to better understand how the virus hijacks cellular machinery to promote tumorigenesis. We also study how KSHV interacts with the host innate immune system. Like other herpesviruses, KSHV establishes lifelong latency in the human population, which means that the virus needs to constantly evade immune surveillance by the host. We study how KSHV is able to modulate host innate immune pathways to its advantage, in order to escape detection and elimination by the host immune system.

At UNC-CH, I also direct the Lineberger Global Oncology program with Dr. Dirk Dittmer. The goal of our program is to investigate cancers that disproportionally affect low-income countries, many of which are viral cancers. Thus far, our achievements have been in cancer diagnosis, basic and translational research, as well as preclinical studies, which represent the cornerstone for treatment. We have implemented essential infrastructures, continue to build capacity, and are developing new approaches to diagnose and treat cancer in these countries.

I am a firm believer in the importance of basic science. It is an indisputable fact that translational applications in medicine have often come from fundamental discoveries, which at the time they were identified had no clinical applicability whatsoever. These basic findings were refined over time and transformed into clinical applications in medicine. This has most certainly been true for viruses in the field of oncology. Viruses led to seminal discoveries that laid the foundation for our current understanding of cancer biology. Today, the study of tumor virology continues to reveal new biological insights into the development of cancer and continues to identify key cellular targets important for tumor initiation, progression, and metastasis. Viruses are also being used as cancer vaccine vectors to train our immune systems to attack the tumor within, and oncolytic viruses are being utilized to treat and lyse human tumors in patients. Thus, it seems we have come full circle. The link between viruses and cancer has been a powerful one throughout history, and the fields of virology and cancer biology will forever remain intertwined.

**Fig 1:**
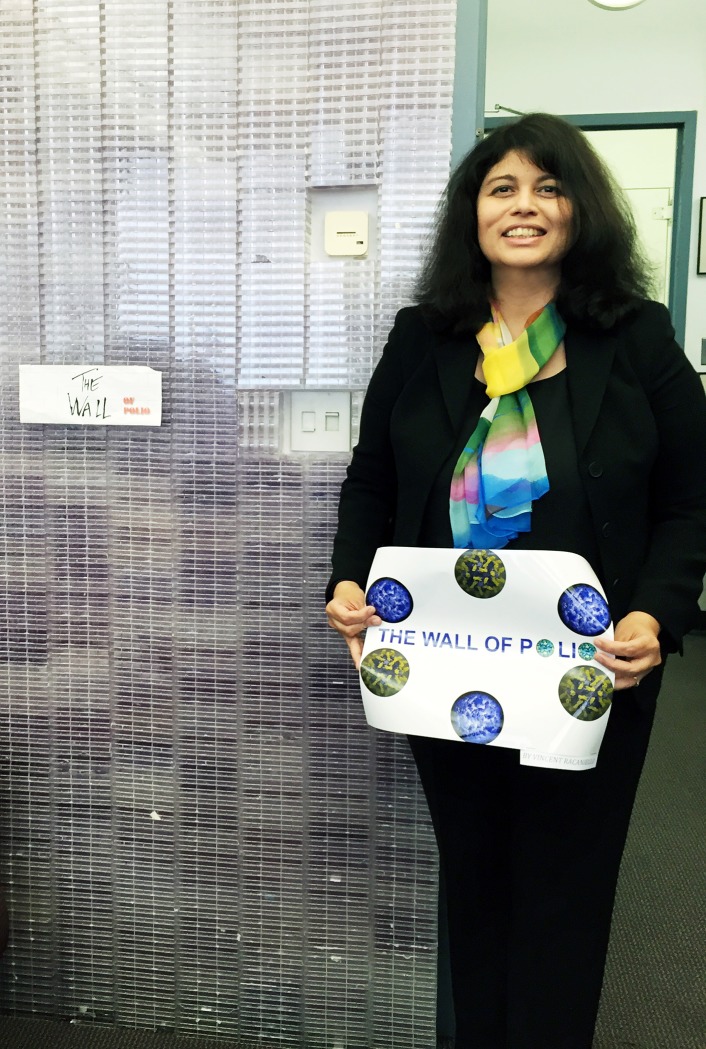
Blossom Damania. Dr. Damania is standing in front of *The Wall of Polio*, a work of art created by Dr. Vincent Racaniello. SV40, an oncogenic DNA tumor virus, was discovered in 1960 as a contaminant of early batches of poliovirus vaccines.

